# Size distribution and relationship of airborne SARS-CoV-2 RNA to indoor aerosol in hospital ward environments

**DOI:** 10.1038/s41598-023-30702-z

**Published:** 2023-03-02

**Authors:** V. Groma, Sz. Kugler, Á. Farkas, P. Füri, B. Madas, A. Nagy, T. Erdélyi, A. Horváth, V. Müller, R. Szántó-Egész, A. Micsinai, G. Gálffy, J. Osán

**Affiliations:** 1 Environmental Physics Department, Centre for Energy Research, Budapest, 1121 Hungary; 2 Department of Applied and Nonlinear Optics, Wigner Research Centre for Physics, Budapest, 1121 Hungary; 3 Department of Pulmonology, Semmelweis University, Budapest, 1085 Hungary; 4 BIOMI Ltd., Gödöllő, 2100 Hungary; 5 Pest County Pulmonology Hospital, Törökbálint, 2045 Hungary

**Keywords:** Environmental sciences, Diseases, Pathogenesis

## Abstract

Aerosol particles proved to play a key role in airborne transmission of SARS-CoV-2 viruses. Therefore, their size-fractionated collection and analysis is invaluable. However, aerosol sampling in COVID departments is not straightforward, especially in the sub-500-nm size range. In this study, particle number concentrations were measured with high temporal resolution using an optical particle counter, and several 8 h daytime sample sets were collected simultaneously on gelatin filters with cascade impactors in two different hospital wards during both alpha and delta variants of concern periods. Due to the large number (152) of size-fractionated samples, SARS-CoV-2 RNA copies could be statistically analyzed over a wide range of aerosol particle diameters (70–10 µm). Our results revealed that SARS-CoV-2 RNA is most likely to exist in particles with 0.5–4 µm aerodynamic diameter, but also in ultrafine particles. Correlation analysis of particulate matter (PM) and RNA copies highlighted the importance of indoor medical activity. It was found that the daily maximum increment of PM mass concentration correlated the most with the number concentration of SARS-CoV-2 RNA in the corresponding size fractions. Our results suggest that particle resuspension from surrounding surfaces is an important source of SARS-CoV-2 RNA present in the air of hospital rooms.

## Introduction

Since the COVID-19 pandemic started, a lot of attention has been drawn towards the emission, transport, infection properties, collection, detection, and particle sizing of the SARS-CoV-2 virus, but still, a lot of challenges and obstacles exist^1–7^. For instance, aerosol sampling, in the environment of patients treated in a hospital must be done in a way that is not disturbing the work of the hospital staff nor the patient’s calm and recovery.

The first studies highlighted that the SARS-CoV-2 spread through direct human-to-human droplet transmission, indirect means such as contact with contaminated objects or surfaces (fomites) and by airborne transmission^8–10^. At the beginning of 2021, the scientific community already emphasized the predominance of airborne mode of transmission^11–13^, which means that floating aerosol particles play an essential role.

According to Birgand et al.^14^, during the first 11 months of the pandemic, 23 studies were carried out studying air contamination by SARS-CoV-2 RNA at hospital sites. The studies focused on the measurement of total aerosol concentration without size fractionation or studied only a few particle size ranges. The measurements yielded surprisingly few positive samples, only 82 out of 471 air samples (17.4%) were positive for SARS-CoV-2 RNA. In intensive care unit settings, the positivity rate was 25.2% (27 of 107 samples), which is significantly higher than the value of 10.7% (39 of 364) characterizing the non-intensive care units. It was also found that the distance between the patients and the sampling point did not play an important role^14^, except for higher distances (more than 2 m)^15^. The mentioned studies found the airborne SARS-CoV-2 RNA concentration between 1 to 113 copies/m^3^^16–21^, except the work of Lednicky et al.^22^, who detected three orders of magnitude higher concentration with a maximum of 94,000 copies/m^3^.

As a result of the developments in aerosol measurement technology and the increase of the sensitivity of PCR methods, and also due to the expansion of opportunities in sampling conditions, more and more aerosol samples were found to be evaluable for SARS-CoV-2 RNA^23^. This provided an opportunity for the scientific community to conduct research oriented towards size fractional aerosol sampling and analysis. Information on the particle size distribution associated the virus can be utilized to inform disease transmission modeling, as well as better understand the inhalability/respiratory deposition pattern of aerosols in exposed individuals. The size of the respiratory droplets/droplet nuclei has an important role in both disease transmission and in airway deposition distribution. Many authors presented numerical models, that described the effect of gravitational settling and evaporation of the droplets in the air^24–28^. Depending on the solute content, small droplets can remain airborne as droplet nuclei for a long time as they can almost completely evaporate during their descent to the ground. Viruses are likely to remain infectious in aerosols for hours, resulting in an increase in the infectious viral air load. According to the review article of Ribaric et al.^29^, several (11) studies were performed to study the size distribution of aerosol particles bearing virus copies in the size range of 0.25–10 µm collected at the patient and non-patient areas of the hospitals. However, none of them was performed in patient areas including size ranges below 250 nm. Overall, it was found that SARS-CoV-2 RNA could be detected in all size ranges, from which the positivity rate is higher in the larger size ranges (2.5–10 µm). The positivity rate of SARS-CoV-2 RNA in the air samples was found to be similar to the overall detection rate of non-size-fractionated airborne particulate matter (PM) samples (approx. 16%).

The primary objective of the present study was to quantify the SARS-CoV-2 RNA prevalence in a hospital ward, and in a specific high-dependency unit (HDU) for a wide size range in numerous fractions from the ultrafine mode (particulate matter of nanoscale size) up to coarse mode (aerodynamic diameter ranging from 2.5 to 10 µm), for many different patient groups allowing statistical evaluation. Based on this, our aim was to determine the characteristic size range of airborne SARS-CoV-2 RNA and its possible change over time due to the disease progresses. By determining the distribution of the particle number concentration with high time resolution, our second aim was to study the effect of particle mass fluctuation due to indoor human activity and the relationship between the number of SARS-CoV-2 RNA copies and the aerosol particle number/mass concentration in hospital wards.

## Methods

### Study design, participants and setting

Measurements were performed in two different hospital wards. At the Pulmonology Hospital of Törökbálint (hereafter Hospital A) only one, whilst at the Department of Pulmonology of Semmelweis University (hereafter Hospital B) three patients were treated at the same time in the studied wards. While in Hospital A a normal pulmonary patient room, in Hospital B a high-intensity non-invasive ventilation unit (HDU) was sampled. Patients treated in a non-invasive ventilation unit require continuous observation and advanced level management, however invasive mechanical ventilation with closed breathing circuit is not used but non-invasive mechanical ventilation can be applied which is able to generate additional airflow around the patient^30^. The aerosol sampling and monitoring instruments were installed as close as possible on the nightstand beside the bed, at the height of the lying patients’ heads at both hospitals. To avoid the disturbing of the patients, the noise generating part of the equipment was placed outside the ward.

In the case of Hospital A, the sampling and monitoring devices were operated at a distance of 3 m from the bed of the patient in a room of 5.0 × 4.8 × 2.5 m^3^ during the entire measurement period (see Figure S1a in Supplementary Material). The patient was hospitalized on the 4^th^ day after the positive test, and he got respiratory support by using a nasal cannula during the measurement period. His medical condition allowed him to move around in the room, and the patient logged his main activities in a diary. Medical treatment, daily cleaning and room ventilation activities were logged by the staff.

In Hospital B HDU the room size was 6.7 × 4.3 × 2.5 m^3^ and the patients were undergoing treatment in all three beds of the ward for varying lengths of time, with varying degrees of ventilator support, including non-invasive ventilation, O_2_ treatment via high flow nasal cannula or reservoir mask. As the patients changed within a short time, there were only 3–4 consecutive days when the same patients were in the ward, but the type of non-invasive respiratory support might have changed according to the improvement or worsening of the clinical condition of the patients (for details see Table S1). Thus, we could define 6 groups of measurement days, which are numbered from B1 to B6 (see Tables 1 and 2). The time of infection and the technique used for respiratory support are summarized in Table S1 (Supplementary Material). In the room at Hospital B, the measurement instruments were placed on a nightstand between the window and the bed on the side opposite the door. The measurement point was 90, 270 and 450 cm far from the three patients (see Figure S1b in Supplementary Material). HDU department is cleaned regularly according to local protocol. All cleaning procedures were performed prior to start of the measurement each day (6:00–6:20 AM).

**Table 1:** Basic information on PM measurements and sampling in hospital wards.

Location	Patient group notation	Measurement period (year 2021)	# of days	# of patients	VOC	# of impactor stages
Hospital A	A1	04 26–05 01	6	1	alpha	7
Hospital B	B1	03 01–03 04	4	3	alpha	5
Hospital B	B2	11 03–11 05	3	3	delta	5
Hospital B	B3	11 09–11 12	4	3	delta	5
Hospital B	B4	11 16–11 19	4	3	delta	5
Hospital B	B5	11 22–11 26*	4	4**	delta	5
Hospital B	B6	12 06–12 08	3	3	delta	5

*No measurement was performed in 11 24 2021.

**One patient was replaced on 11 24 2021.

**Table 2:** Rate of positivity and quantifiability for each set of size-fractionated PM samples.

Patient group	Measurement date	Positivity rate (number of samples with N2 above detection limit/number of all samples)	Quantifiability rate (number of samples ≥ 10 copies for N2 positive/number of all samples)
A1	4 26 2021	6/7	2/7
	4 27 2021	3/7	3/7
	4 28 2021	2/7	2/7
	4 29 2021	3/7	3/7
	4 30 2021	5/7	5/7
	5 1 2021	6/7	4/7
B1	3 1 2021	4/5	3/5
	3 2 2021	4/5	4/5
	3 3 2021	3/5	3/5
	3 4 2021	3/5	3/5
B2	11 3 2021	1/5	0/5
	11 4 2021	0/5	0/5
	11 5 2021	0/5	0/5
B3	11 9 2021	2/5	1/5
	11 10 2021	5/5	0/5
	11 11 2021	4/5	1/5
	11 12 2021	5/5	3/5
B4	11 16 2021	3/5	3/5
	11 17 2021	2/5	2/5
	11 18 2021	4/5	4/5
	11 19 2021	4/5	4/5
B5	11 22 2021	1/5	0/5
	11 23 2021	1/5	0/5
	11 25 2021	3/5	0/5
	11 26 2021	4/5	1/5
B6	12 6 2021	4/5	0/5
	12 7 2021	3/5	0/5
	12 8 2021	5/5	4/5

During the sampling period, the variant of concern (VOC) of SARS-CoV-2 was alpha VOC for Hospital A and included periods of alpha and delta VOC for Hospital B. It is important to note that during delta VOC, most patients have already received their baseline vaccinations. This could affect the shedding of patients as reported in Refs.^31,32^.

In Hospital A only one patient, whilst in Hospital B nineteen patients were studied. This allowed us to study both the time evolution of virus concentration for a single patient and differences of virus concentration resulting from multiple patients with different degrees of disease severity.

### Sampling and data collection

An in-house built May-type cascade impactor^33^ was used to sample size-fractionated aerosol particles on presterilized gelatin disc filters (Sartorius, Göttingen, Germany) as collecting substrate. The easily dissolvable gelatin filers have suitable performance for collecting viruses for PCR analysis^34^. Two versions of the impactor were used, a 7 stage basic version, which has aerodynamic cut-off diameters of 16, 8, 4, 2, 1, 0.5 and 0.25 μm for stages 1 to 7, respectively at 20 L/min sampling flowrate, whilst the 9 stage in-house developed extended version is suitable for collecting further size fractions with cutting diameters of 0.18 and 0.07 μm (stages 8 and 9 respectively)^35^. Cut-off diameters below 1 µm were verified through a comparison measurement using an aerosol spectrometer^36^. Total particle mass agreed well with reference filter sampling and size distributions were found to be similar to those obtained using a commercial Dekati impactor^37^. The sampling time was 8 h in both cases, during daytime including the main time for medical interventions. No sampling was performed on stages 1 and 2 since particles larger than 10 μm aerodynamic diameter are beyond the range of PM_10_. Sampling periods are summarized in Table 1. In Hospital B, 22 sample sets were collected (5 stages each, altogether 110 samples), while in Hospital A, only 6 sample sets (7 stages each, altogether 42 samples).

Simultaneously with the 8-h impactor samplings, an optical particle counter (GRIMM PAS 1.109) was used to measure the indoor particle mass concentration of PM_1_, PM_2.5_ and PM_10_ (particle matter with an aerodynamic diameter less than 1, 2.5 and 10 µm, respectively) with a time resolution of 1 min. Sampling by the impactor provides information on the concentration and size fractions of virus laden particles without any information on the concentration and size distribution of all the aerosol particles from the ward. Sampling by the OPC provides useful information on the number and size of all particles. Simultaneous sampling by the impactor and OPC allowed the analysis of correlation between the size distribution of the aerosol and SARS-CoV-2 RNA concentration in aerosol particles for each patient group. Since the OPC data are obtained at a high time resolution, and the values are influenced also by the outdoor air quality, first a preprocessing of the PM mass concentration data measured by OPC was applied for each 8-h sampling period.

### Detection and quantification of SARS-CoV-2

Size-fractionated PM samples were collected onto presterilized gelatin filters (Sartorius) at stages 3 to 7/9 of the impactor. The gelatin filters were removed and placed in a clean and sterile holder and were forwarded within 12–72 h for virus testing, which is an acceptable timeframe for specimen transportation and storage^38^. The gelatin filters were dissolved in 600 µl RAV1 buffer of the NucleoSpin RNA Virus kit (Macharey-Nagel, Düren, Germany), and the isolation of viral RNA was carried out following the kit's protocol. This included incubation at 70 °C for 5 min, adding 600 μL ethanol and loading it on the MN NucleoSpin Viral RNA isolation spin-columns. A centrifugation step of 1 min at 8000 g followed, and the washing steps included three washing steps (1st wash: 500 μL RAW buffer 1 min at 8000 g, 2nd wash 600 μL RAV3 buffer 1 min at 8000 g, 3rd wash 200 μL RAV3 buffer 5 min at 11,000 g). The final elution step included adding 60 μL prewarmed (70 °C) TE buffer, incubating it on the column for 1–2 min, and carrying out a centrifugation at 11,000 g for 1 min^39^.

The standard curve was derived from the ATCC Heat Inactivated 2019 Novel Coronavirus VR-1986HK (ATCC, Manassas, USA) by isolating RNA with the above-described method and preparing ten-fold dilutions. The samples were analyzed according to the methods by Refs.^40,41^, see details in Supplementary Material (Table S2). Sensitivity, specificity and validity tests of the method are discussed in detail in Ref.^41^. Only samples with at least 10 copies of the N2 sequence for a given aerosol size gelatin were used for statistical analysis.

Sampling with the May-type cascade impactor was performed at nearly similar daytime periods of 8 h to reach the appropriate detection limit of the PCR test. According to Ribaric et al.^29^, among sampling methods used at different hospital sites, impactors were found to yield one of the lowest mean concentrations of detected SARS-CoV-2 RNA. The detection limit of the above-detailed methodology is similar to that reported in other studies (around 2 copies/sample^16,42^), while the quantification limit was 10 copies/sample. The results for airborne concentrations of the SARS-CoV-2 N2 gene are expressed in copies/m^3^, taking into account the 9.6 m^3^ sampled air volume (operating flow rate of 20 L/min during 8 h). Thus, in the present study, the quantification limit for airborne concentrations is 1.04 copies/m^3^ for each size fraction.

### Ethical approval

The measurements of the multicenter observational, non-interventional case-only study were completed based on a protocol approved by the Semmelweis University Regional and Institutional Committee of Science and Research Ethics (SE RKEB) (approval no 219/2020). All methods were performed in accordance with the relevant guidelines and regulations. All voluntarily participating patients signed written consent after being informed both orally and in written form.

## Results and discussion

Several studies investigating the aerosol concentration and elemental composition in hospital wards^43–46^ highlighted the importance of the outdoor aerosol concentration influencing the indoor concentration of the particulate matter. It has long been known that outdoor particles are the main contributors to indoor particles in hospital environments^47^, and a linear relationship exists between indoor and outdoor PM concentrations^48,49^. As direct outdoor PM measurement were not performed, correlation between the measured indoor and outdoor PM concentrations available from the closest urban reference station was studied. Therefore, first, the connection between indoor and outdoor aerosol load was investigated in the present study for both measurement locations. The highest correlation was found between the minimum PM_2.5_ concentrations measured indoors and those reported at the closest urban environmental reference station in Budapest (see Figure S2 in Supplementary Material). Taking into account the 8-h sampling period each day, the Pearson correlation coefficients were *r* = 0.94 and *r* = 0.87 for Hospitals A and B, respectively. Thus, it can be assumed that the minimum concentration measured indoors can be considered as a lower estimate, practically a baseline that is dependent on the outdoor aerosol concentration. The concentration increment above the baseline is characteristic of the indoor events and sources of PM. Therefore, the outdoor environmental effect was minimized by considering the PM concentration increments for each 8-h sampling period, by subtracting the respective minimum PM concentrations from all 1-min PM values, as preprocessing of the OPC data. In the next step, we examined the characteristics of the high time-resolution size-fractionated PM parameters measured by the optical counter (trend, maximum, mean, fluctuation of concentration increment) for each day. In the final step, a Pearson correlation analysis of aerosol parameters and SARS-CoV-2 RNA concentration in each size range was performed for each patient group.

### Particle number size distribution trends

There are significant differences between the two hospital sites in terms of environmental and internal conditions. Hospital A is located in a suburban area, while Hospital B is in downtown Budapest. The impact of the different sites is clearly reflected in the PM concentrations and their daily trends (see Fig S2). During the whole measurement campaign, the daily outdoor minimum PM_2.5_ concentration was between 3.8 and 6 µg/m^3^ at Hospital A, whereas generally higher values were detected at Hospital B for the same variable (5.4–29.6 µg/m^3^). In addition, only one patient was treated in Hospital A during the sampling period, whose medical condition allowed more active forms of free movement (getting out of bed, telephony, and so on). By contrast, in the downtown ward (Hospital B), three patients were treated in the respiratory HDU setting using different aerosol-generating therapeutic devices and the patients were not able to leave their beds. Although high-flow nasal oxygen (HFNO) respiratory supports are aerosol-generating procedures, the emission rates of this equipment are highly dependent on the respiratory activities and the settings. Depending on the health status of the patients, we can assume that the direct contribution of the respiratory support units to aerosol concentration is not significant (~ 1.5–5 particles/cm^3^)^50,51^. However, in the case of HDU, due to the frequent activity of medical staff and the often displacement of the respiratory mask, which results in high airflow inside the ward, air turbulence-related dispersion of airborne particles can play a very important role^52^. Therefore, besides the passive room ventilation (through the adjacent control room for Hospital B), the resuspension generating processes basically determined the quantity and typical size distribution of aerosol concentration in the ward. In general, the detected size distributions showed two maxima observed in size ranges below 1 μm and around 10 μm, which is typical for ambient aerosols in urban environments. In addition, especially at Hospital B, an additional small maximum at around 4 μm was observed occasionally, presumably related to indoor activity.

**Figure 1:**
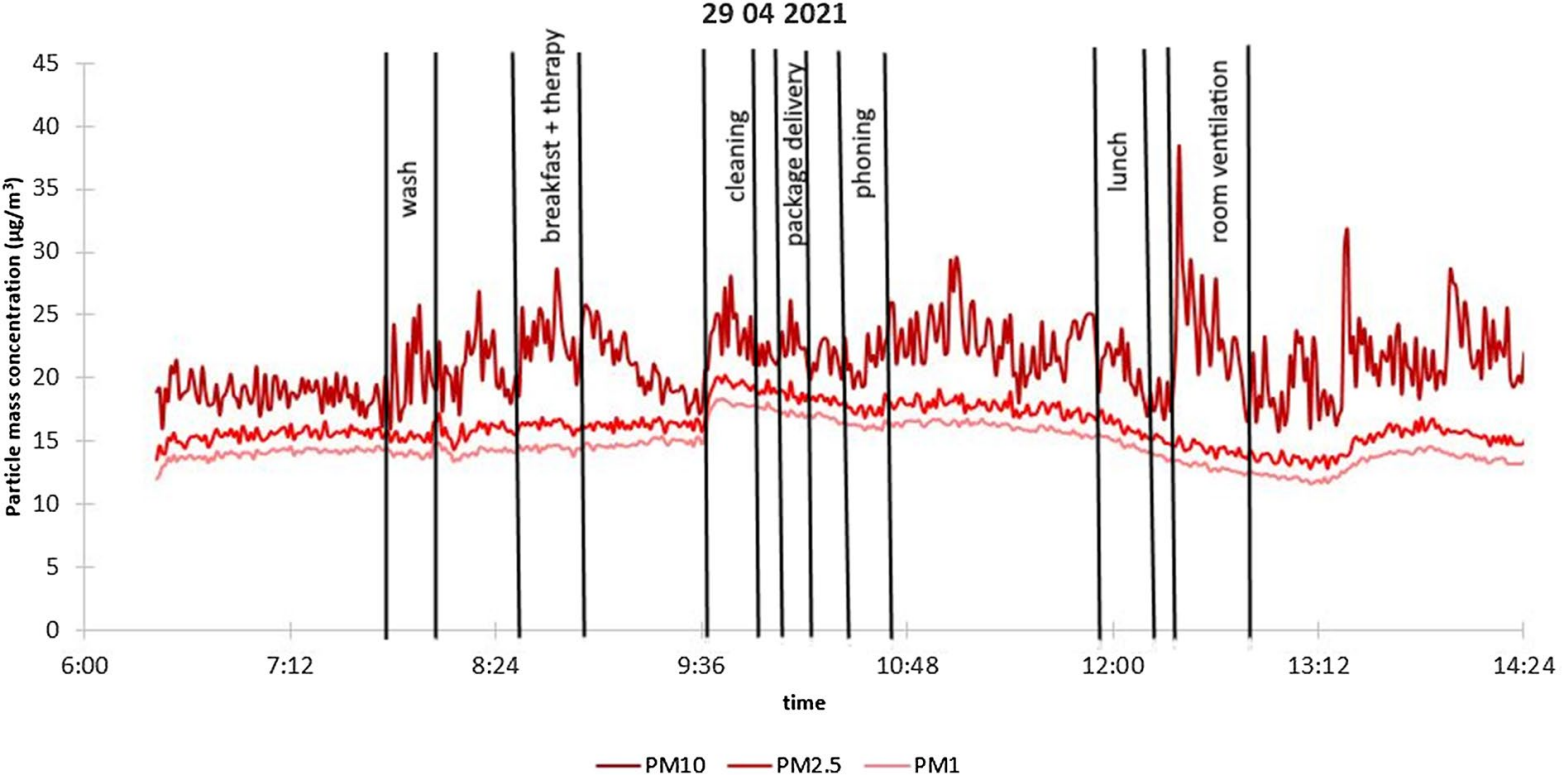
Event log and particle mass concentration trends at Hospital A, 04 29 2021.

Based on an itemized event log available in the case of Hospital A, the daily time trends of PM concentrations could be studied in detail in relation to indoor human activity. It is important to note that short events, such as coughing, were not registered. It was found that for all activities, an increment in PM_10_ concentration can be assigned to each movement, while for smaller size ranges (PM_2.5_ and PM_1_) an increase can only be observed during certain events (room ventilation, cleaning). O’Neil et al.^53^ found typically just a few µg/m^3^ total mass concentration increase due patient’s activity (like eating, bathing, tidying), while Nagy et al.^54^, highlighted that each (even similar) activities could cause very different particle number increments in the different size ranges. Health care professional (HCP) activities related to physician and/or nurse visits for medical diagnostic examinations and nursing care are among the most important events causing the increase in the number of submicron particles. By the same token, activities related to full patient care, but especially bedding, have contributed significantly to the rise of larger particles’ concentration in the air. Moreover, increases in supermicron particle concentration were associated with the number of HCPs and the duration of the activity, while submicron particles increased with all activities^54^. All these demonstrates that particle size distribution is a function of the activity type. Our results show that the magnitude of increments linked to different events was diverse. While in the case of room cleaning, the increase of PM_10_ concentration was up to 60 µg/m^3^, the effect of bathing led only to an increment of 8–13 µg/m^3^. Generally, 7–9 significant events could be registered in one day (see Fig. 1.).

In the case of Hospital B, although three patients were treated in parallel, the patients were not active, and room ventilation was indirect, so much fewer—typically 1–4—events were detected each day indirectly based on the OPC data. However, the effect of these events was found to be much larger at this site. The increase in PM_10_ concentration was found to be up to 180–315 µg/m^3^. As this is a HDU, this is in line with the high-intensity activity of HCPs.

### Size distribution of SARS-CoV-2 RNA copies

Based on the results of published studies so far, it can be generally concluded that the positivity rate of SARS-CoV-2 RNA surface and indoor air samples collected from the patient’s environment is similar^23,29^. In addition to direct contact, deposition of airborne particles also contributes to surface contamination. At the same time, from the aspect of airborne transmission, it is important to know the abundance of each particle size range that the virus is likely to be attached to. To date, only a few studies have been performed investigating the size distribution of infectious aerosol. In the work of Liu et al.^16^, a size distribution of 6 size ranges was obtained as a result of a long 16-h sampling. Stern et al.^42,55^ performed successful measurements for three size ranges with a sampling time of 48 h, while Moharir et al.^56^ presented measurements with a much larger sampling flow rate (100 L/min) and a much shorter sampling time (10 min) suitable only for establishing the fact of positivity. In the present study, several 8-h samples collected using a 20 L/min flow rate are discussed for 5/7 size fractions.

The number of SARS-CoV-2 virus copies was found to be highly variable in the studied size fractions. In the case of Hospital A, within the 6 sample sets, 25 out of the 42 samples (59.5%) were determined to be positive of which 19 (45.2%) samples were quantifiable for the SARS-CoV-2 virus (see details in Table 2).

**Figure 2:**
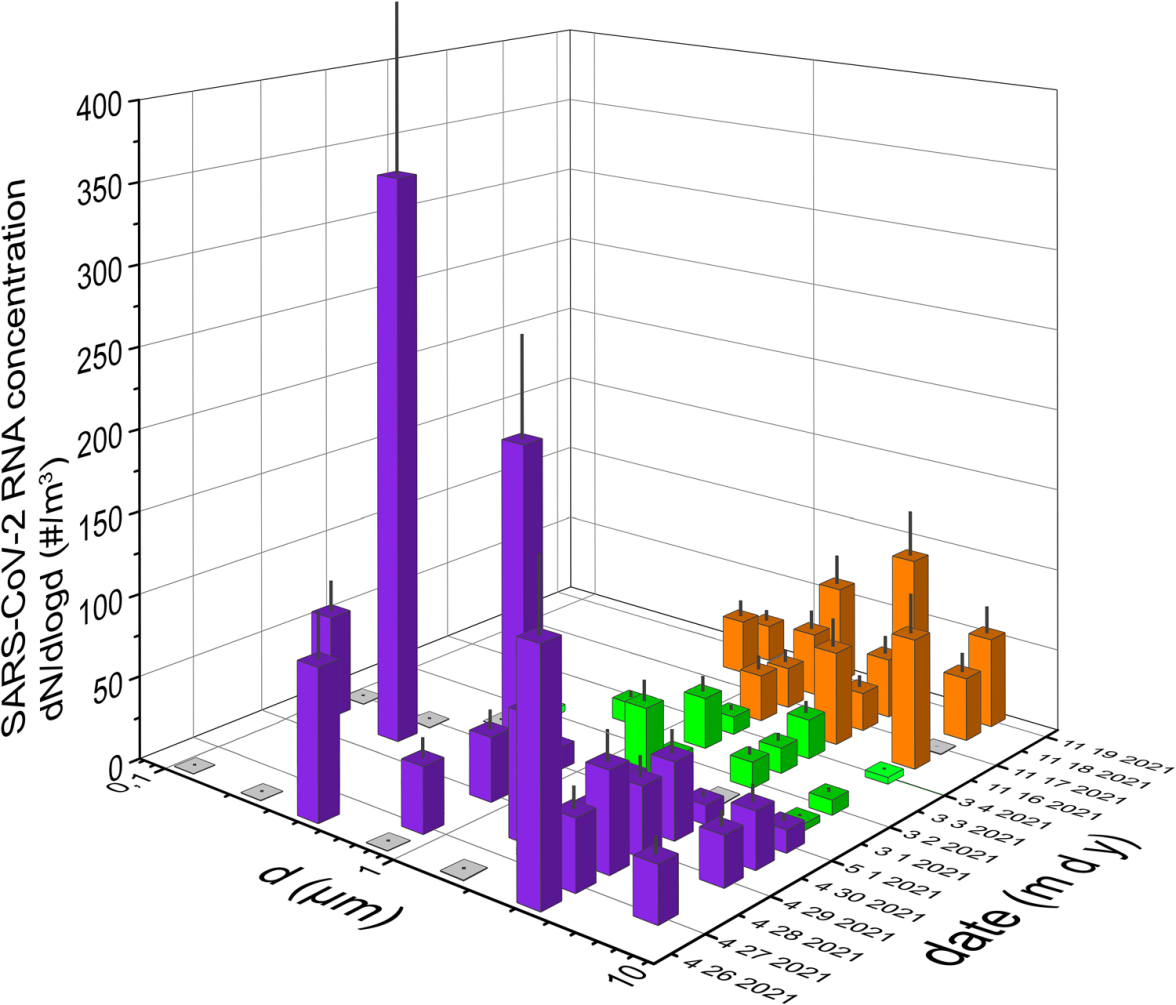
Size distribution of the number of SARS-CoV-2 RNA copies for A1 group (purple) and B1 (green) and B4 (orange) two patient groups. The relative standard deviation of PCR is estimated as 31%, indicated on the bars. Transparent bars refer to positivity but below the quantification limit.

Size variation of the number of copies shows a bimodal distribution in all cases except for one day (04 28 2021), however the locations of maxima vary in size from day to day. The average of SARS-CoV-2 N2 gene number concentration ranged between 18 and 184 copies/m^3^ for the measurement period (see Fig. 2) between 0.07 and 8 µm. A significantly higher value was detected on 04 30 2021, when an extremely high number of SARS-CoV-2 RNA was found in the 230 nm and 800 nm mode size ranges. Based on the event log, it can be concluded that no extraordinary event occurred during that day. The patient was more and more active, however his PCR test was still positive, which might have cause the significant virus release detected.

In the case of Hospital B, 65 out of the 110 samples (59.1%) was found to be positive of which 35 (32.8%) was quantifiable, besides a narrower size range (250 nm–8 μm see details in Tables 1 and 2). For this size interval the total number of SARS-CoV-2 varied in the range of 0.9–59.9 copies/m^3^ on an 8-h daily average covering the most active 8 h of patient care at the HDU. As the patients changed within a short time we could define 6 groups of measurement days, however we could obtain quantitative information for only two groups (marked with green (B1) and orange (B4), see Fig. 2), where the number of SARS-CoV-2 RNA copies could be determined for all days and the entire size range. Based on these measurement results, it was found that an unimodal size distribution is typical in all cases, of which the maximum varies within the 1–4 μm diameter range. In case of these 3 sample sets all samples were found to be SARS-CoV-2 RNA positive in the 2–4 μm size range, however the number of SARS-CoV-2 RNA copies was found to be below the quantification limit in the size range of 250–500 nm. Also, no clear time trend in size distribution was observed during any of the 4-day periods.

For the remaining four groups (B2, B3, B5 and B6), the number of SARS-CoV-2 RNA copies were below the detection limit in a major proportion of the collected samples. Mostly samples corresponding to the size fraction of 0.5–1 μm showed values above the detection limit, suggesting that if highly virus-laden particles are present, they are more likely to exist on particles in this size range. According to Ribaric et al.^29^ summarizing the few available measurement results, it is not possible to establish a clear size distribution of the SARS-CoV-2 RNA copy number, as the summarized results of the collected studies show that the positivity rate in hospital rooms and waiting rooms is similar (~ 30%) for both 1–4 µm and < 1 µm size ranges (Table 2). In the current study, based on larger number of samples with a higher size resolution, we can confirm that the maximum of the size distribution typically varies in the size range of 0.5–4 µm. In the work of Stern et al.^55^ it was also reported that SARS-CoV-2 RNA positive samples could be found in a wide size range, however it was most frequent in the 2.5–10 µm size range. It should be noted that the differing results of the A1 group could be the consequence of high infectious SARS-CoV-2 alpha VOC as vaccination was low at that time. Tan et al.^31^ demonstrates a decrease in the probability of transmission in previously exposed/vaccinated individuals, which would suggest decreased viral shedding. The difference could also be due to a range of other possible factors, such as the wide interpersonal variability in shedding reported in COVID-19 patients^32^. Among the many factors that determine the exact location of the maximum in the size distribution (atmospheric conditions, indoor air quality, patients’ virulence, indoor human activity, etc.), our aim was to examine the effect of particle load and fluctuations. It should be mentioned the fact that some of the patients may have not released detectable amounts of virus under the conditions tested, thus they were no longer infectious during hospitalization (see Table S1), which suggests that the patients’ virus emission rate has a very high personal variety, in accordance with the results reported in Ref.^31,57^. Considering this, and the fact that three patients could have released the virus in parallel, the discussion on the number of days since symptom onset in relation with airborne virus load is not straightforward. However, in case of Hospital B, where 18 patients were examined in total, it was found that the virus release was much lower for those patients who had spent more than 20 days in the hospital since the onset of symptoms (63% of days studied).

**Figure 3:**
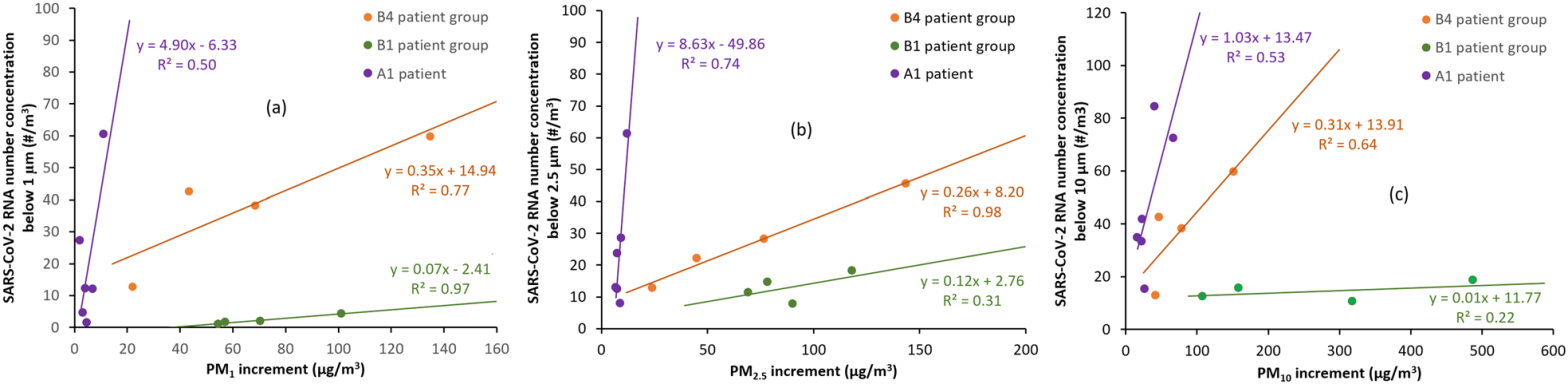
PM vs SARS-CoV-2 number concentrations for three measurement periods (A1, B1 and B4 patient groups) for the (**a**) below 1 µm, (**b**) below 2.5 µm and (**c**) below 10 µm size ranges and the fitted linear regression lines.

To investigate the significance of the aerosol load status of the wards, a correlation analysis was performed between the PM mass concentration increment and the number of SARS-CoV-2 RNA N2 copies in each size range. Those periods were evaluated for which at least the 80 percent of samples were quantifiable by PCR (i.e., A1 and B1 and B4 groups). Among the statistical values of daily PM mass concentration trends (min, max, mean, increment etc.) medical staff and patient activity related highest concentration increment was found to show the strongest correlation for the size ranges studied (PM_10_, PM_2.5_ and PM_1_). SARS-CoV-2 copies were summed for all impactor stages within the size range studied (i.e. stages (9,8,)7,6 for PM_1_; all stages for PM_10_). Correlation coefficients of linear regression for the three periods (patient groups) and the studied size ranges are summarized in Table 3, while the corresponding scatter plots are shown in Fig. 3.

**Table 3:** Coefficients of linear regression for size fractionated SARS-CoV-2 and PM mass concentration variables for three group of patients.

Patient group	Coefficients of linear regression *R*^2^			Slope of linear fit		
	PM_1_	PM_2.5_	PM_10_	PM_1_	PM_2.5_	PM_10_
A1 (7 stage data)	0.67	0.79	0.21	15.71	26.57	1.52
A1 (5 stage data)	0.50	0.74	0.53	4.90	8.63	1.03
B1	0.97	0.30	0.21	0.07	0.26	0.31
B4	0.77	0.98	0.64	0.35	0.16	0.01

The most important difference between the two sites in terms of sampling is that the extended version of May impactor was used at Hospital A, which allowed us the collection of particles below 250 nm. As the number of SARS-CoV-2 in this size range was remarkably high on certain days it should be emphasized that the investigation of this size range is also recommended. This is also confirmed by the correlation analysis results, since a more accurate conformity is found (rows 1 and 2 of Table 3) if the smallest size ranges (stages 8 and 9) are taken into account. To the best of our knowledge our study is the first to provide data for this important particle size range.

According to different investigators, the median/mean equivalent spherical diameter of SARS-CoV-2 (without the spikes) was between 60 and 140 nm^16,58–62^. The size depended on the sample collection (e.g. dissection) and preparation (freezing, fixation etc.) method and also on the measurement technique (e.g. atomic force microscopy, transmission electron microscopy). There was also an inherent inter-individual variability of the virion size, but the diameter of the investigated individual virions regularly fell into the 50–160 nm size range. As in our study on the last two stages of the extended impactor the sampled particles were 180–250 nm and 70–180 nm large, it is a plausible question whether the detected RNA fragments originated from virions contained in very small, eventually dried out droplets, unattached virions or merely virus fragments.

Exhaled droplets range in diameter from 0.01 and 1000 µm depending on the physical activity, health status, generation mechanism and the site of origin, among others^63^. The smallest particles are formed in the smallest airways due to the mechanism of elasto-capillary instability related to airway reopening. Though the median mass is represented by particles in the size range of 0.7 to 1 µm^64^, exhaled droplets of 100–200 µm are also common and these particles represent a significant particle fraction of the total number concentration^65^. After exhalation, the size of these droplets can further decrease by the evaporation of volatile content, thus at least theoretically it is possible that the RNA copies detected on the lowest stages (especially the second lowest stage) of the impactor originate from entire virions. Obviously, it cannot be excluded that the particles impacting here contained or were only virion fragments containing the N2 sequence.

A strong linear relationship was found for the smaller (PM_1_ and PM_2.5_) size ranges, while it was moderate for the greater (total, under 10 µm) size range. In general, the aerosol sources in hospital wards can basically be (i) the mixing of outdoor particles due to room ventilation^47–49^, (ii) the emission of people by breathing and speaking, etc.^63–65^, (iii) resuspension as a result of their or staff activities^54^, and (iv) the direct and indirect particle emission of machines (respirator apparatus) operating in the room^30,50,51^. As the effect of outdoor sources was eliminated, and the quantity of particles emitted during breathing by the hospital staff wearing masks and by patients even with respiratory support^50,51^ was found to be orders of magnitude smaller than the concentration fluctuations due to human activities, it can be stated that the resuspension effect of inside movements are determinant for indoor PM concentration increase. Although patients are the primary source of airborne SARS-CoV-2 RNA, these virus laden particles are transmitted to the surfaces by direct contact or by sedimentation. Direct contact might be responsible for SARS-CoV-2 RNA identified on the patients’ bed, nightstand and patients’ phone as we detected it in surface swabs in Hospital B. As resuspension of all particles are significant it can be concluded that the medical stuff activities (such as movement and treatment) could result in an excess of virus-laden aerosols in the indoor atmosphere. It is worth noting that the virus detection method used in this work could not distinguish between viable and non-viable viruses/virions.

The slope of the linear regression lines (Fig. 3) was generally much higher for Hospital A than for Hospital B, while in case of PM_2.5_ it was in the same magnitude at Hospital A for different patient groups. This suggests that under identical site layout and measurement setup, the viral content of the indoor air is proportional to the increase in particle number concentration caused by indoor activities, taking into account that the characteristics of viral emission of each individual person is highly variable^32,66^.

## Conclusion

In the present study, high numbers of size-fractionated aerosol samples collected in hospital wards were analyzed to investigate the potential viral load of aerosol particles over a wide range of particle sizes. As a result, we could also obtain information about the ultrafine size range that, to the best of our knowledge, had not been studied so far. The particle size distribution associated the virus is important, since the lung deposition probability has a minimum at around 400 nm, alveolar deposition increases with decreasing particle diameter ^67^. As fine (PM_1_) particles can reach the alveolar surface, they might cause direct alveolar infection with highly contagious pathogens.

The detected SARS-CoV-2 RNA positivity rate of nearly 60% and the quantification rate of air samples of nearly 50% indicate that sampling and analysis were successful in the 70 nm–8 µm size range. In addition, the length of the sampling period allowed for statistical evaluation of the collected data. The detected size distributions were typically unimodal in the 0.25–10 µm range, and significant quantities of SARS-CoV-2 RNA were detected even in the sub-300 nm size range. This suggests that the enveloped SARS-CoV-2 virus might even be able to spread in the air without being attached to a carrier aerosol particle. The location of the maximum in the size distribution was different for different patient groups, and there was no temporal trend, suggesting that the viral load of the hospital room is not primarily determined by the duration of the patient’s positivity.

Statistical evaluation of size-fractionated particle number concentrations and SARS-CoV-2 RNA number concentrations indicated that the number of virus-containing particles increased with the increased aerosol particle number due to human activity. This is likely to be the consequence of the observed large proportion of infected surfaces, as discussed in numerous studies^29^. Moreover, the results suggest that the virus RNA number concentrations in the air are determined by indoor aerosol sources and do not appear to be dependent on total indoor PM concentrations.

Surface disinfection, therefore, plays a key role not only in preventing infection through contact but also in reducing the airborne spread of infectious particles.

The significant fraction of positive samples in the < 250 nm size range can be important also from the perspective of face masks. It is well known that the filtering efficiency of the masks is related to their capacity to filter out particles as large as or larger than 300 nm. Virtually, these masks could not be effective in the case of viruses found in the < 250 nm size fraction. However, it has been demonstrated that impaction and interception characterizing the > 300 nm particles are not the only filtering mechanisms and smaller particles are also well filtered due to diffusional and electrostatic attraction mechanisms^68^.

During the COVID pandemic, the use of standardized ventilator devices was not allowed in the sampled wards, so these data might be different in non-pandemic times. Nevertheless, it is likely, that the current findings are valid for a number of other respiratory pathogens. Therefore, present results may serve as valuable inputs when designing hospital wards, but also when planning the accommodation and treatment of infected patients.

## Limitations of the study

One of the weaknesses of our study is that sampling was carried out over an 8-h period to ensure the collection of a critical amount of samples without the disruption of the ward’s activity. As the RNA copy results were integrated to this 8-h period, the relative impact of individual activities in the wards could not be assessed. In addition, we did not have sufficient and evaluable samples with high N2 copies to evaluate the infectivity, as the virus could be deactivated during sampling with the cascade impactor and PCR is not giving information on viability of the virus.

An additional limitation was the fact that sampling spanned over two different VOC periods with significant changes in the vaccination status of patients, associated with shorter recovery, implying different degrees of virus emissions^69,70^, however, this supposition needs further investigations.

## Supplementary Information

Supplementary Information.

## Data Availability

The data related to the present study can be obtained from the corresponding author J. Osán (osan.janos@ek-cer.hu) upon personal request.
