# Supplementary material for: Size distribution and relationship of airborne SARS-CoV-2 RNA to indoor aerosol in hospital ward environments
Source: Sci Rep. 2023 Mar 2;13:3566. doi: 10.1038/s41598-023-30702-z (PMC9980870; doi:10.1038/s41598-023-30702-z)
Supplement: Supplementary file 1 — Supplementary Information. [file 41598_2023_30702_MOESM1_ESM.pdf]

Supplementary Material

Size distribution and relationship of airborne SARS-CoV-2 RNA to indoor aerosol in hospital ward environments

V. Groma[1], Sz. Kugler[1], Á. Farkas[1], P. Füri[1], B. Madas[1], A. Nagy[2], T. Erdélyi[3], A. Horváth[3], V. Müller[3], R. Szántó-Egész[4], A. Micsinai[4], G. Gálffy[5] and J. Osán[1*]

[1] Environmental Physics Department, Centre for Energy Research, Budapest, 1121, Hungary
[2]Department of Applied and Nonlinear Optics, Wigner Research Centre for Physics, Budapest, 1121, Hungary
[3]Department of Pulmonology, Semmelweis University, Budapest, 1085, Hungary
[4]BIOMI Ltd., Gödöllő, 2100, Hungary
[5] Pest County Pulmonology Hospital, Törökbálint, 2045, Hungary

[*]Corresponding author: J. Osán, e-mail: osan.janos@ek-cer.hu

Table S1. Measurement days and treatment of patients on different beds and duration of positivity. Colors define the different patient’s groups.

Location	Measurement date	# of patients	Type of respiratory support			Time since first symptoms (day)			time since positive test (day)			time since negative test (day)		
			Bed A	Bed B	Bed C	Bed A	Bed B	Bed C	Bed A	Bed B	Bed C	Bed A	Bed B	Bed C
Hospital A	4/26/2021	1	nasal cannula	-	-	5	-	-	5	-	-			
Hospital A	4/27/2021	1	nasal cannula	-	-	6	-	-	6	-	-			
Hospital A	4/28/2021	1	nasal cannula	-	-	7	-	-	7	-	-			
Hospital A	4/29/2021	1	nasal cannula	-	-	8	-	-	8	-	-			
Hospital A	4/30/2021	1	nasal cannula	-	-	9	-	-	9	-	-			
Hospital A	5/1/2021	1	nasal cannula	-	-	10	-	-	10	-	-			
Hospital B	3/1/2021	3	nasal cannula	NIV	HFNO	20	7	8	13	7	6			3
Hospital B	3/2/2021	3	nasal cannula	NIV	HFNO	21	8	9	14	8	7			4
Hospital B	3/3/2021	3	nasal cannula	NIV	nasal cannula	22	9	10	15	9	8	0		5
Hospital B	3/4/2021	3	NIV	NIV	NIV	23	10	4	16	10	2	1		

Hospital B	11/3/2021	3	HFNO	HFNO	HFNO	12	19	23	9	15	9			
Hospital B	11/4/2021	3	NIV	HFNO	res. mask	13	20	24	10	16	10			
Hospital B	11/5/2021	3	NIV	HFNO	res. mask	14	21	25	11	17	11			
Hospital B	11/9/2021	3	NIV	res. mask	HFNO	18	14	9	15	4	4			0
Hospital B	11/10/2021	3	NIV	HFNO	HFNO	19	12	10	16	7	5			1
Hospital B	11/11/2021	3	NIV	HFNO	HFNO	20	13	11	17	8	6			2
Hospital B	11/12/2021	3	NIV	HFNO	HFNO	21	14	12	18	9	7			3
Hospital B	11/16/2021	3	res. mask	HFNO	NIV	8	15	13	12	15	3			
Hospital B	11/17/2021	3	res. mask	NIV	NIV	9	16	14	13	16	4			
Hospital B	11/18/2021	3	res. mask	NIV	NIV	10	17	15	14	17	5			
Hospital B	11/19/2021	3	res. mask	NIV	NIV	11	18	16	15	18	6			
Hospital B	11/22/2021	3	res. mask	res. mask	HFNO	14	21	19	18	21	9			
Hospital B	11/23/2021	3	NIV	res. mask	HFNO	15	22	20	19	22	10			
Hospital B	11/25/2021	3	HFNO	res. mask	HFNO	16	23	21	3	23	11		0	0
Hospital B	11/26/2021	3	NIV	res. mask	HFNO	17	24	22	4	24	12		1	1
Hospital B	12/6/2021	3	NIV	NIV	nasal cannula	9	3	3	5	3	3			
Hospital B	12/7/2021	3	NIV	NIV	nasal cannula	10	1	4	6	1	4			
Hospital B	12/8/2021	3	NIV	NIV	nasal cannula	11	2	5	7	2	5			

Table S2. Primer-probe sets used for RT-PCR assays

Target gene	Primer/Probe	Sequence[a]	Final concentra tion	Ref
Nucleo-capsid (N1)	2019-nCoV_N1-F	5’-GACCCCAAAATCAGCGAAAT-3’	200 nM	[1]
	2019-nCoV_N1-R	5’-TCTGGTTACTGCCAGTTGAATCTG-3’	200 nM	
	2019-nCoV_N1-P	5’-FAM-ACCCCGCATTACGTTTGGTGGACC-BHQ1-3’	200 nM	
Nucleo-capsid (N2)	2019-nCoV_N2-F	5’-TTACAAACATTGGCCGCAAA-3’	200 nM	
	2019-nCoV_N2-R	5’-GCGCGACATTCCGAAGAA-3’	200 nM	
	2019-nCoV_N2-P	5’-FAM-ACAATTTGCCCCCAGCGCTTCAG-BHQ1-3’	200 nM	
Envelope (E)	E_Sarbeco_F	5’-ACAGGTACGTTAATAGTTAATAGCGT-3’	400 nM	[2]
	E_Sarbeco_R	5’-ATATTGCAGCAGTACGCACACA-3’	400 nM	
	E_Sarbeco_P1	5’-FAM-ACACTAGCCATCCTTACTGCGCTTCG-BHQ1-3’	200 nM	

a FAM: 6-carboxyfluorescein; BHQ1: Black Hole Quencher-1.

Figure S1. Layout of hospital ward at Hospital A. (a) and Hospital B (b) Box no. 1-3 marks the location of patients and 4 marks the location of measurement equipment

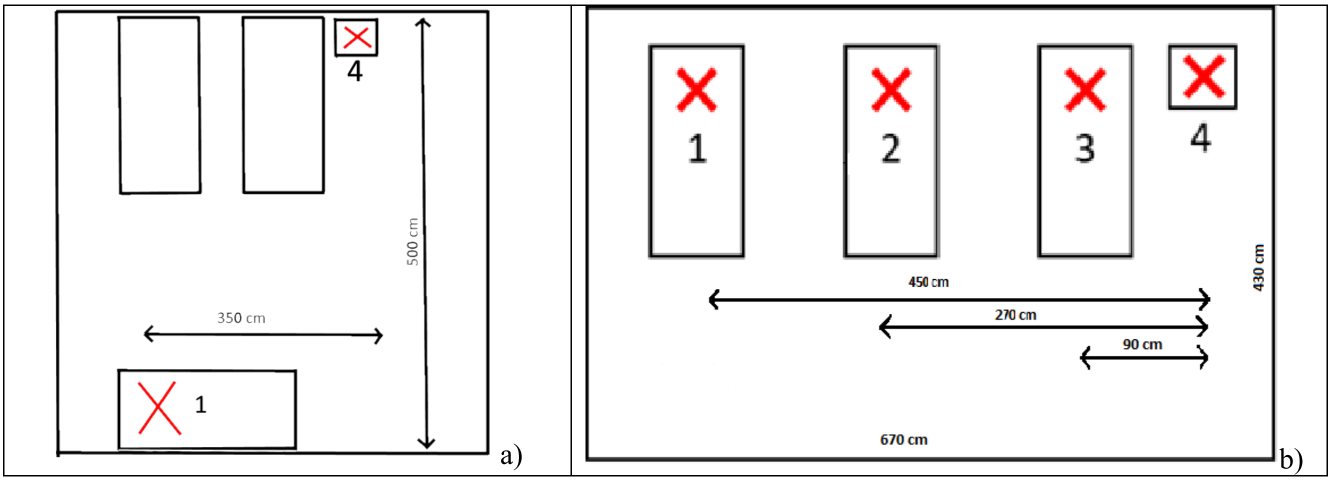

Figure S2. Daily minimum values of measured $PM_{2.5}$ concentrations during the measurement periods at Hospital sites and reference station in downtown Budapest

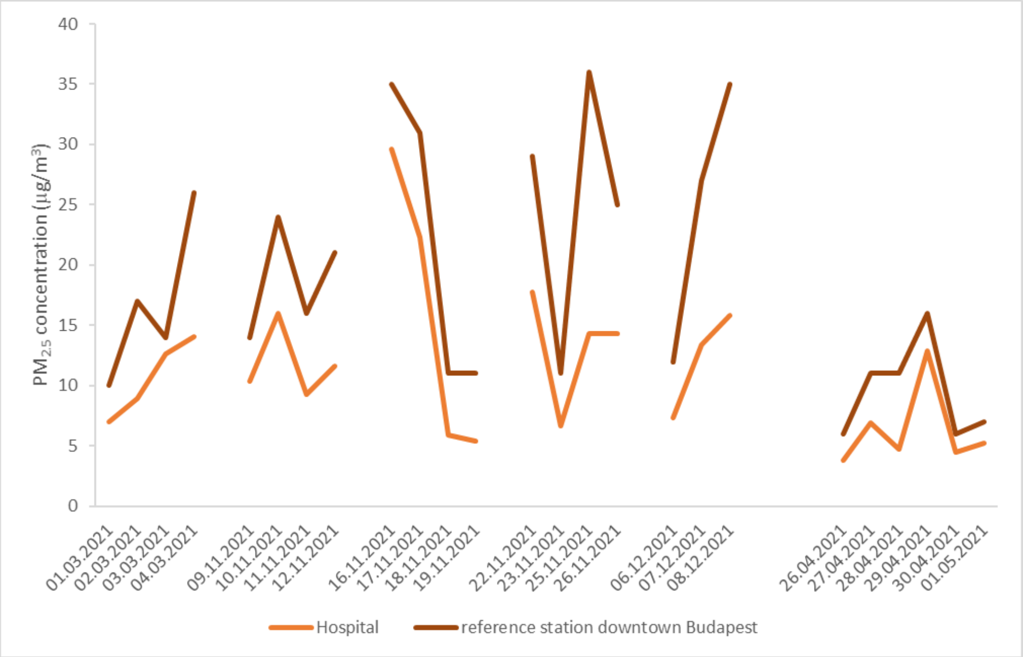

References

[1] "Real-time RT-PCR Primers and Probes for COVID-19 | CDC." https://www.cdc.gov/coronavirus/2019-ncov/lab/rt-pcr-panel-primer-probes.html (accessed Jun. 24, 2022).

[2] V. M. Corman *et al.*, "Detection of 2019 novel coronavirus (2019-nCoV) by real-time RT-PCR," *Eurosurveillance*, vol. 25, no. 3, p. 2000045, Jan. 2020, doi: 10.2807/1560-7917.ES.2020.25.3.2000045.
